# A new seven-axis robotic-assisted total hip arthroplasty system improves component positioning: a prospective, randomized, multicenter study

**DOI:** 10.1038/s41598-024-63624-5

**Published:** 2024-06-02

**Authors:** Run Tian, Xu Gao, Ning Kong, Xinghua Li, Yiyang Li, Jian Wang, Yongping Cao, Zhanjun Shi, Kunzheng Wang, Pei Yang

**Affiliations:** 1 Department of Bone and Joint, Xi’an Jiaotong University Second Affiliated Hospital, No.157, Xiwu Road, Xincheng District, Xi’an, 710004 Shaanxi China; 2 Xi’an Honghui Hospital, Xi’an, China; 3 Department of Radiology, Xi’an Jiaotong University Second Affiliated Hospital, Xi’an, China; 4 Department of Orthopedics, Southern Medical University Nanfang Hospital, Guangzhou, China; 5 Department of Orthopedics, Peking University First Hospital, Beijing, China

**Keywords:** Total hip arthroplasty, Robot, Clinic trial, Randomized controlled trials, Diseases

## Abstract

This study compared the radiologic and clinical outcomes of a new seven-axis robotic-assisted total hip arthroplasty (THA) and conventional THA. Hundred and four patients were randomly assigned to two groups—the robotic-assisted THA group (RAS group) and the conventional THA group (CON group). The preoperative and postoperative Harris Hip score (HHS), acetabular inclination, anteversion, femoral offset, and leg length discrepancy (LLD) were compared. During the follow-up, no patients had any complications that could be associated with the use of the robot. The proportion of acetabular cups in the safety zone was significantly higher in the RAS group than that in the CON group. The two groups had significantly different mean absolute difference of inclination and anteversion. There was no significant difference in the postoperative HHSs, changes in HHSs, femoral offset, and lower limb length between the two groups. The seven-axis robotic-assisted THA system is safe and effective, and leads to better acetabulum cup positioning compared to conventional THA. The improvements observed in the HHS, LLD, and femoral offset in the RAS group were similar to those in the CON group.

*Clinical trial registration time*: 19/05/2022.

*Clinical trial registration number*: ChiCTR2200060115.

## Introduction

Total hip arthroplasty (THA) is the gold standard for the treatment of most late-stage hip diseases, including femoral head necrosis, hip osteoarthritis, and congenital hip dysplasia. A large number of patients undergo THA every year in China, and this number is increasing quickly every year^1^. Acetabular cup stability plays a crucial role in the implant survival rate, patient functionality, and overall THA success rate^2,3^. An abnormal acetabular cup position leads to an unstable hip joint. In the previous decades, Lewinnek suggested a “safe zone” as the criterion for acetabular cup positioning^4^. However, some previous studies showed that the “safe zone” was not applicable for all hips^5^. For cups located in the “safe zone,” a risk of hip dislocation and instability is also present requiring more complex revision surgery, which would increase financial burden and pain for the patient^6^. Therefore, personalized implantation requires thorough preoperative planning and accurate execution of the preoperative plan intraoperatively.

Recently, the robotic-assisted THA system has been introduced for clinically performing THA^7^. This is a new technology that firstly uses computed tomography data from the patient’s lower limb to formulate a preoperative plan including three-dimensional planning for orientation and components size assessment, then uses a robotic arm to resect the bone and insert the THA components according to the preoperative plan. Several robotic-assisted total knee arthroplasty systems, including CORI, VELYS, ROSA, and Mako, have been implemented clincially^8–10^. Among these, Mako can also be applied for THA. Previous studies have shown that robotic-assisted THA offers more precise implant placement, better reconstruction of offset, and smaller differences in leg length discrepancy (LLD)^11,12^. However, the clinical application of these robotic systems is limited by their technical complexity, insufficient versatility, and increased operative time. Additionally, traditional six-axis robots taking Mako as represent may not be able to complete their motion trajectory in more complex operating room environments. Increasing the freedom of robotic-assisted systems promises to solve these problems. Sun et al.^13^ showed that a seven-axis robot-assisted total hip arthroplasty system could be mastered with a learning curve of only 13 cases, which is better than the traditional six-axis robot (14–35 cases)^14^. However, There is currently a lack of large multicenter studies assessing it’s effectiveness, safety and clinical outcomes after a seven-axis robot-assisted THA system.

Presently, JianJia is the first seven-axis robotic hip surgery systems to be approved for THA by the National Medical Products Administration of China. In our previous research, the new seven-axis robotic syetem was safe and effective in total knee arthroplasty^15,16^. But as mentioned earlier, THA requires higher demand of surgical accuracy and safety. Therefore, more researches are needed to demonstrate the safety and accuracy of the seven-axis robotic-assisted system applied to THA, as well as whether it still advantageous compared to traditional surgery. It is beneficial for the development and improvement of robotic-assisted THA systems, particularly in China.

**Figure 1:**
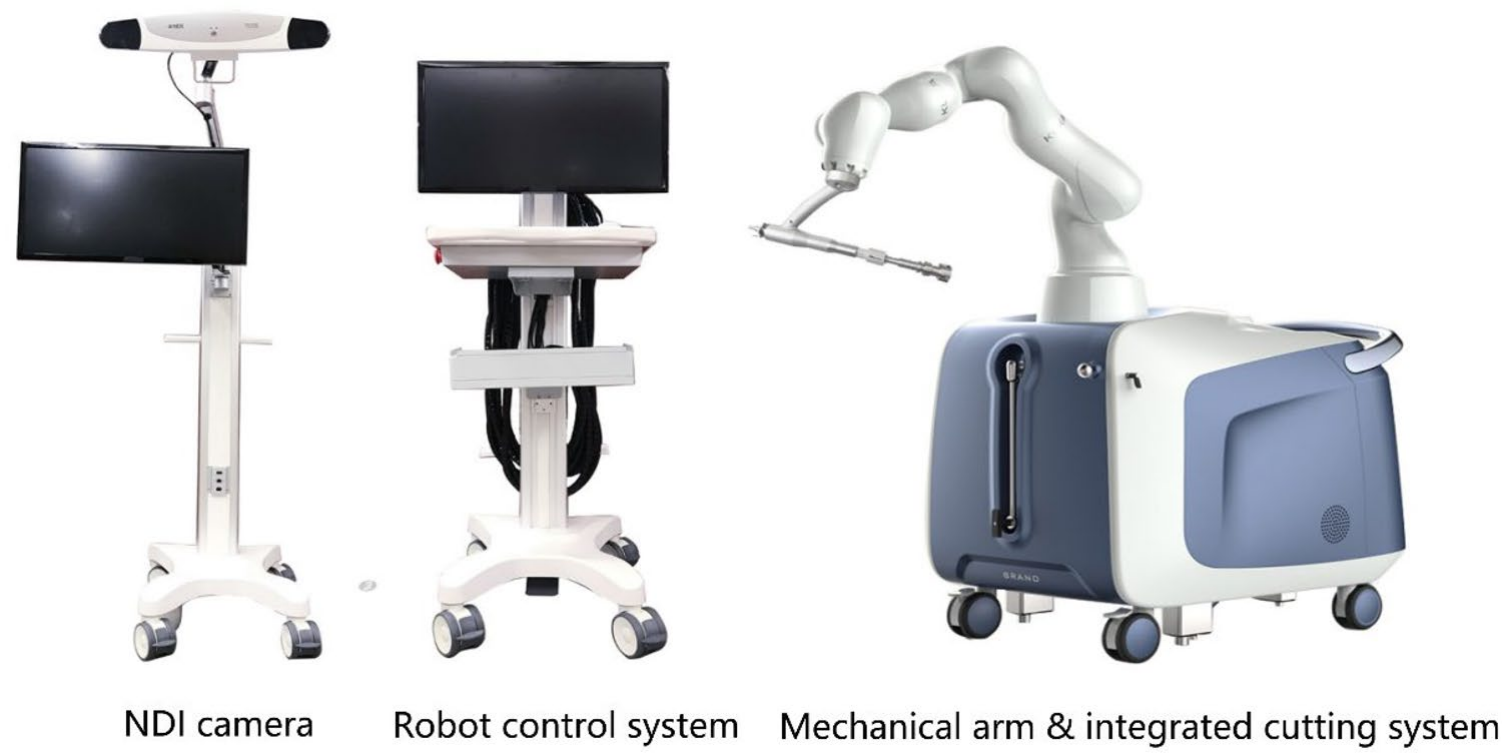
The surgical platform of the “JianJia” robot-assisted THA system.

The National Healthcare Security Administration of China has classified orthopedic robots, and the robotic-assisted THA system used in this study is classified as one that “participated in the surgical operations.” This new robotic-assisted THA system (“JianJia,” Hang Zhou Jianjia Robot Technology Co. Ltd) comprises three parts—a trolley and mechanical arm, central control computer, and signal receiver (Fig. 1). CT data from the patient’s lower limb was used to formulate a preoperative plan and perform three-dimensional planning for orientation. The seven-axis robotic arm was applied to resect the bone and place the components, which was theoretically in line with the principle of human arm movement. The current study is a randomized, multicenter, two-arm, parallel-group controlled trial of the “JianJia” robotic-assisted THA versus conventional THA to explore the effectiveness and safety of this new seven-axis robotic-assisted THA system clinically.

## Materials and methods

### Study design

The study was a randomized, multicenter, parallel controlled trial that was approved by the Institutional Review Board of the Second Affiliated Hospital of Xi’an Jiaotong University (NO. 2020001, DATE: 2020-1-08). The study was registed on Chinese Clinical Trial Registry, and register number is ChiCTR2200060115. All methods were performed in accordance with the relevant guidelines and regulations of World Health Organization (WHO). Three centers in different regions of China participated in this study, including the Second Affiliated Hospital of Xi'an Jiaotong University, the First Hospital of Peking University, and the Southern Hospital of Southern Medical University. All patients provided written informed consent.

### Randomization

Each center competed for enrollment, adopted the block random method, used an interactive web response system to assign the patients who agreed to participate in the study to one of the groups—the conventional techniques group (CON group) and the robotic-assisted system group (RAS group) at a 1:1 ratio, and then submitted the results into the central randomized system. Researchers intervened through the results of the randomized allocation.

### Sample size calculation

Previous studies have reported that 43–62% of patients who underwent conventional THA had the acetabular cup placed in the safe zone^17,18^. It was estimated that the proportions of patients with the acetabular cup in the safe zone after THA (acetabular cup placement accuracy rate) in the CON group and RAS group would be 70% and 93%, respectively. The significance level was set at 0.025, and the test efficacy was set at 0.8. The minimum number of cases required for the test was 82, and considering a 20% dropout rate, the minimum number of cases required was 104.

### Patient recruitment

Between July 2020 and March 2021, 104 patients were recruited. Follow-up was completed in 98 patients—47 patients underwent robotic-assisted THA and 51 underwent conventional THA. The inclusion criteria were as follows: a. aged 18–80 years; b. had surgical indications for THA, including primary or secondary hip osteoarthritis, femoral head necrosis, femoral neck fracture, and congenital hip dysplasia; c. undergoing primary THA; and d. the patient or the legal representative understands the study and agrees to sign an informed consent form approved by the ethics committee, thus agreeing to participate in this study. The exclusion criteria were as follows: a. a body mass index (BMI) of > 35 kg/m^2^; b. the presence of neuromuscular dysfunction that could lead to postoperative hip instability or gait abnormalities, such as paralysis, myolysis, or abductor weakness; c. the presence of active infection; d. the presence of severe osteoporosis, metabolic bone disease, or radioactive bone disease around the hip joint; e. a history of allergies to one or more of the implanted materials; f. the presence of coagulation dysfunctions and uncorrectable bleeding tendency; g. the presence of pregnancy or planning a pregnancy during the study period; h. participation in clinical trials of other drugs or medical devices within 30 days prior to screening, poor in compliance, and unable to complete the study as required. All operations were performed by experienced surgeons for the conventional and robotic-assisted groups at the three hospitals. All patients who underwent THA with the robotic-assisted or conventional operations underwent clinical and radiologic assessment.

### Surgical procedures

**Figure 2:**
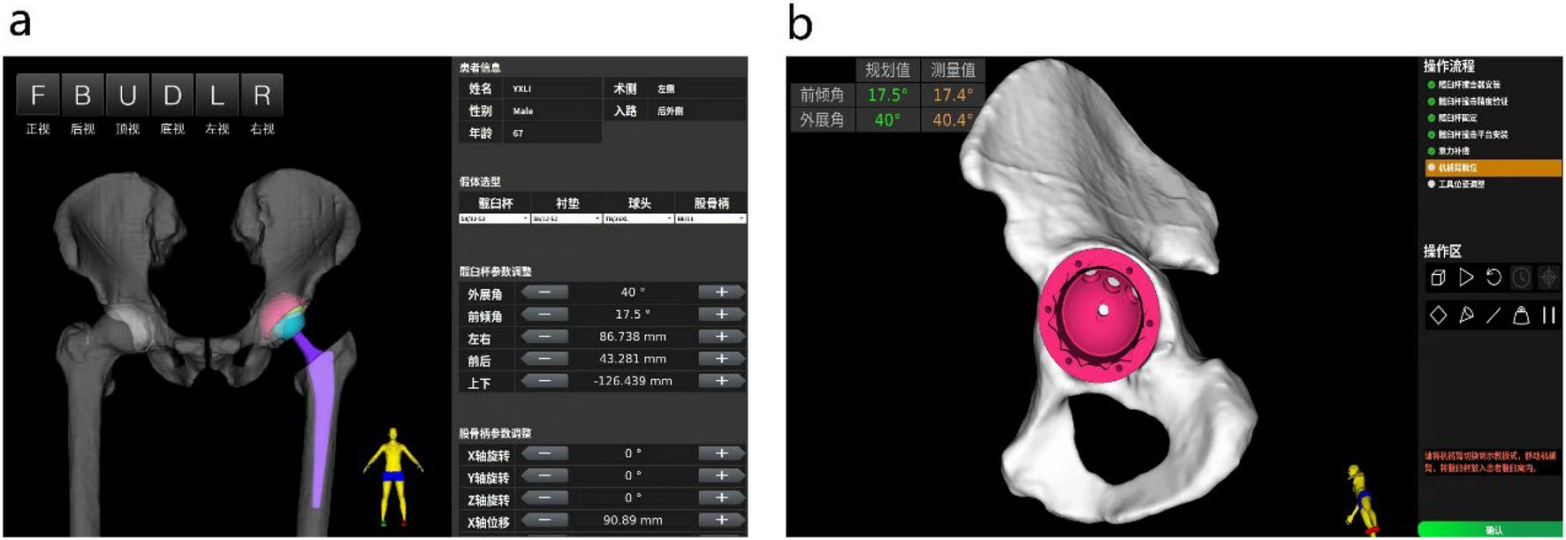
The preoperatively planned position and actual placement during surgery by the robot-assisted THA system.

Prior to initiating the study, all surgical personnel were trained in cadaver handling. Preoperative full-length plain CT scan data of the patient's lower limbs (thickness: 0.625 mm) were obtained, and the data were imported into the preoperative planning module of the robotic-assisted THA system to obtain a preoperative planning scheme (Fig. 2).

All patients underwent THA via a posterolateral approach under general anesthesia. A signal matrix was installed 4 cm superior to the anterior superior iliac crest on the surgical side to transmit the pelvic position to the signal receiver. After the exposure of the acetabulum, the hip joint was dislocated posteriorly and femur osteotomy was performed 1 cm superior to the lesser trochanter. Next, the medullary cavity was prepared and filled with gauze strips. Subsequently, the acetabulum was exposed, the labrum and a portion of the joint capsule were removed, and the acetabulum position was registered with a probe; the bias was < 0.1. Next, the acetabulum was rasped and filed using a mechanical arm under the limits of the defined inclination and anteversion. After filing, the soft tissue in the acetabulum was cleaned again, the acetabular prosthesis was installed with assistance from the mechanical arm, and the prosthesis position was verified. The femoral prosthesis was installed and the hip joint was reduced to confirm stability. No drainage device was placed in any patient. All patients underwent an anteroposterior X-ray of both hip joints and a full-length CT scan of both lower limbs on the 2nd to 3rd postoperative day.

### Clinical outcome evaluation

Clinical data, including age, sex, height, body weight, and other general information, were collected preoperatively. An independent investigator collected other clinical information preoperatively and postoperatively and recorded it in the database. The primary evaluation index was the rate of acetabular cup placement accuracy (inclination: 30–45° and anteversion: 10–25°). Safety was assessed based on the rate of device-related adverse events, dislocations, and device defects. The secondary evaluation indexes included: Harris Hip score (HHS), deviation of anteversion and inclination, LLD, and femoral offset. Preoperative clinical scores were collected on the day before surgery and postoperative clinical scores were measured at a minimum interval of 3 months postoperatively by an independent investigator.

### Radiologic outcome evaluation

**Figure 3:**
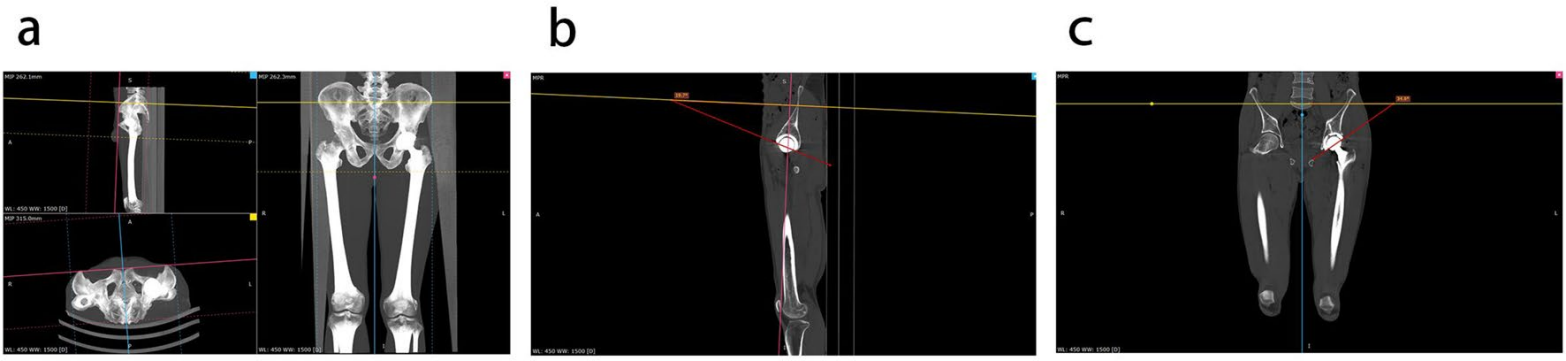
Radiologic outcome evaluation. (**a**) Adjust the image with the anterior pelvic plane defined by the two anterior superior iliac spines and the pubic symphysis. (**b**) Measurement of the anteversion. (**c**) Measurement of the Inclination.

Preoperative (within 7 days before the surgery) and postoperative (2–3 days after the surgery) radiologic data were obtained by two independent investigators. An anteroposterior radiograph and CT scan of the entire lower limb were performed. The anteversion, inclination, offset, and LLD were measured based on the CT scan data according to the methods mentioned in Pankaj’s study^19^. The images were rectified using fixed anatomical markers at different locations. Simply, in the multi-plane reconstruction image, we used the maximum density projection technique to adjust the image in the coronal view with the connection of the bilateral anterior superior iliac spine as the reference line. We take the anterior pelvic plane as the reference plane. In axial images, the line of the anterior iliac superior spine was used as the datum line to adjust the image. In sagittal images, the line between the anterior superior iliac spine and the anterior symphysis pubis was used as the datum line to correct the angle of the anterior and posterior hip bones. The axial, coronal, and sagittal views were restored to the original thin layer image, and the angle between the acetabulum edge and the two reference lines was measured. Murray DW defined anteversion as the angle between the acetabular axis and the coronal plane, defined inclination as the angle between the longitudinal axis and the acetabular axis when it is projected on to the coronal plane^20^. By mathematical conversion, anteversion is the angle between the acetabular margin and the datum line in a sagittal position. Inclination is the angle between the acetabular margin and the datum line in a coronal position (Fig. 3).

### Statistical analysis

All data were exported from the electronic medical record system, analyses were performed using IBMSPSS software (version: 22.0, SPSS Inc. Headquarters, USA) and the statistical packages R (The R Foundation; http://www.r-project.org; version 3.4.3) and EmpowerStats (www.empowerstats.com; version: 3.0, X&Y Solutions Inc.). Continuous variables are displayed as mean ± standard error of mean (SEM) and categorical variables are presented as frequencies (percentages). Pearson’s χ^2^ test or Fisher’s exact test was used for qualitative data, and the Kruskal–Wallis test was used for quantitative data. Logistic regression analysis was used to identify possible confounders and independent factors that could affect safe zone ratio. The differences were considered as statistically significant at a *P* value < 0.05.

## Results

### Results of demographic data

The mean follow-up period of all patients was 3 months. There was no significant difference in age, sex, and BMI between the CON and RAS groups (Table 1). This study included patients with osteoarthritis, femoral head necrosis, rheumatoid arthritis, congenital hip dysplasia, and femoral neck fracture. During the entire follow-up, no complications, such as infections and dislocations, occurred in any case. There was no statistical difference in the composition ratio of the different types of diseases between the two groups. The mean preoperative HHS of all patients was 53.93 ± 15.65, and there was no statistical difference between the two groups in the overall HHS and the proportion of patients with different scores (Table 2).

**Table 1:** Demographic data.

Index statistics	Sum (N = 98)	RAS group (N = 47)	CON group (N = 51)	*P* value
Age (years)				
Average value ± Standard Deviation	56.06 ± 11.14	55.33 ± 12.10	56.74 ± 10.25	0.704
Min, Max	21, 77	26, 77	21, 76	
Gender, n (%)				
Male	58 (59.2)	30 (63.8)	28 (54.9)	
Female	40 (40.8)	17 (36.2)	23 (45.1)	0.369
BMI (kg/m^2^)				
Average value ± Standard Deviation	24.11 ± 3.31	23.64 ± 3.03	24.55 ± 3.52	0.197
Min, Max	17.4, 34.3	18.3, 31.1	17.4, 34.3	

*BMI* body mass index.

**Table2:** Statistical description of present medical history, and HHS scores.

Index Statistics	Sum (N = 98)	RAS group (N = 47)	CON group (N = 51)	*P* value
Primary disease type, n (%)				0.754
Osteoarthritis	32 (32.65)	15 (31.91)	17 (33.33)	
Femoral head necrosis	46 (46.94)	20 (42.55)	26 (50.98)	
Rheumatoid arthritis	11 (11.22)	7 (14.89)	4 (7.84)	
Femoral neck fracture	6 (6.12)	3 (6.38)	3 (5.88)	
Congenital hip dysplasia	3 (3.06)	2 (4.26)	1 (1.96)	
HHS overall score				
Average value ± Standard Deviation	53.93 ± 15.65	53.72 ± 14.68	54.12 ± 16.64	0.902
Min, Max	12, 93	12, 82	15, 93	
HHS overall score group, n (%)				0.630
≥ 85 points	2 (2.04)	0 (0)	2 (3.92)	
60–84 points	29 (29.59)	14 (29.79)	15 (29.41)	
< 60 points	67 (68.37)	33 (70.21)	34 (66.67)	

*HHS* Harris hip score.

### Results of inclination and anteversion of acetabulum cup compared to preoperation planing

The average difference between the actual postoperative measured inclination and the preoperatively planned inclination was − 1.45 ± 5.72 (2.11 ± 6.76 in the CON group and − 0.74 ± 4.27 in the RAS group), and there was no significant difference between the two groups. The average difference between the actual postoperative measured anteversion and the preoperatively planned anteversion was 7.54 ± 9.67 (9.97 ± 11.36 in the CON group and 4.93 ± 6.60 in the RAS group), and these values were not significantly different between the two groups. To further accurately assess the difference between the actual and the preoperatively planned acetabular cup positions, we compared the absolute value of the difference in the inclination and anteversion. The average difference of absolute value between the actual postoperative measured inclination and the preoperatively planned inclination was 4.62 ± 3.63 (5.71 ± 4.12 in the CON-group and 3.45 ± 2.58 in the RAS-group), and these values were not significantly different between the two groups. The average difference in the absolute value between the actual postoperative measured anteversion and the preoperatively planned anteversion was 9.29 ± 8.00 (12.01 ± 9.12 in the CON group and 6.33 ± 5.23 in the RAS group), and this difference was not significantly different between the two groups. The overall proportion of the acetabular cups located in the safe zone was 65.31% (50.98% in the CON group and 80.85% in the RAS group), indicating a statistically significant difference between the two groups (*P* = 0.002) (Table 3).

**Table 3:** Statistical description of inclination and anteversion of acetabulum cup when compared to preoperation planing.

Index Statistics	Sum (N = 98)	RAS group (N = 47)	CON group (N = 51)	*P* value
Difference of inclination				
Average value ± Standard Deviation	− 1.45 ± 5.72	− 0.74 ± 4.27	− 2.11 ± 6.76	0.240
Min, Max	− 15.90, 14.40	− 9.50, 10.10	− 15.90, 14.40	
Abs of DI				
Average value ± Standard Deviation	4.62 ± 3.63	3.45 ± 2.58	5.71 ± 4.12	0.002
Min, Max	0.00, 15.90	0.00, 10.10	0.30, 15.90	
Difference of anteversion				
Average value ± Standard Deviation	7.54 ± 9.67	4.93 ± 6.60	9.97 ± 11.36	0.009
Min, Max	− 10.50, 31.50	− 10.50, 22.10	− 10.50, 31.50	
Abs of DA				
Average value ± Standard Deviation	9.29 ± 8.00	6.33 ± 5.23	12.01 ± 9.12	0.000
Min, Max	0.10, 31.50	0.30, 22.10	0.10, 31.50	
Proportion in the safe zone, n (%)	64 (65.31)	38 (80.85)	26 (50.98)	0.002

*DI* difference of inclination, *DA* difference of anteversion.

### Results of HSS in follow-up when compared to baseline

There was a statistically significant increase in the average HHS at 3 months postoperatively (36.07 ± 15.28) compared to the preoperative values. The HHS of the RAS group at 3 months postoperative ranged from 40.0 to 99.0 points, with an average score of 89.85 ± 7.16 points. Compared with the baseline data, the postoperative HHS in RAS group showed a significant increase of 36.13 ± 13.35 points. The HHS in the CON group ranged from 39.0 to 98.0 points, with an average of 90.14 ± 8.19 points. Compared with the baseline data, the postoperative HHS in CON group showed a significant increase of 36.02 ± 17.35 points. There was no significant difference in the HHS and the HHS improvement between the two groups (Table 4).

**Table 4:** Statistical description of HSS overall score of participants in follow-up when compared to baseline.

Follow up statistics	Sum (N = 98)	RAS group (N = 47)	CON group (N = 51)	*P* value
Baseline				
Average value ± Standard Deviation	53.93 ± 15.65	53.72 ± 14.68	54.12 ± 16.64	0.902
Min, Max	12, 93	12, 82	15, 93	
POD 12weeks ± 10 days				
Average value ± Standard Deviation	90.00 ± 7.68	89.85 ± 7.16	90.14 ± 8.19	0.855
Min, Max	68.00, 100.00	75.00, 100.00	68.00, 100.00	
Relative Change of Baseline	36.07 ± 15.28	36.13 ± 13.35	36.02 ± 17.35	0.971

*POD* postoperative days.

### Results of femoral offset and LLD postoperative

The mean absolute value of the postoperative femoral offset difference between the two groups was 0.59 ± 0.91 (0.63 ± 1.20 in the CON group and 0.54 ± 0.42 in the RAS group), and these values were not significantly different between the two groups.

The mean value of the absolute LLD difference postoperatively was 0.92 ± 0.71 (0.96 ± 0.73 in the CON group and 0.89 ± 0.70 in the RAS group), and these values were not significantly different between the two groups (Table 5).

**Table 5:** Statistical description of femoral offset and LLD postoperative.

	Sum (N = 98)	RAS-group (N = 47)	CON-group (N = 51)	*P* value
Abs of femoral offset difference				
Average value ± Standard Deviation	0.59 ± 0.91	0.54 ± 0.42	0.63 ± 1.20	0.631
Min, Max	0.00, 7.76	0.06, 1.63	0.00, 7.76	
Abs of LLD				
Average value ± Standard Deviation	0.92 ± 0.71	0.89 ± 0.70	0.96 ± 0.73	0.618
Min, Max	0.00, 3.30	0.00, 3.00	0.10, 3.30	

*LLD* leg length discrepancy.

### Results of the safe zone ratio in logistic regression model

To adjust for the influences of sex and BMI on the safe zone ratio, we constructed a regression model. The results showed that the RAS group achieved a higher safe zone ratio compared to the CON group in the multivariate analysis model (odds ratio: 4.09, 95% confidence interval: 1.62–10.37, *P* = 0.003) (Table 6).

**Table 6:** Correlation between different groups and safe zone ratio in logistic regression model.

	Crude model		Adjusted model	
	OR (95%CI)	*P* value	OR (95%CI)	*P* value
CON-group	Reference		Reference	
RAS-group	4.06 (1.63, 10.09)	0.003	4.09 (1.62, 10.37)	0.003

Crude model: we did not adjust for any covariates.

Adjusted Model: we adjusted for sex and BMI.

*OR* odds ratio, *CI* confidence interval.

## Discussion

THA has been effectively employed in managing severe hip joint disorders. Accurate intraoperative cup and stem position determine the clinical outcomes of THA. Although proper preoperative planning can ensure a successful surgery, prosthesis placement intraoperative usually varies widely depending on the surgeon's experience and the anatomical landmarks^21^, which may mar the usefulness of preoperative planning. The emergence of robot assisted systems provides greater possibilities for consistency between preoperative planning and intraoperative implementation. But its safety and accuracy are still the focus of people's attention. At the same time, the traditional six-axis robot is not enough to adapt to complex clinical application scenarios due to limitations such as long learning curve, operating room space and operation fluency. People have tried to solve these problems by improving the degree of freedom of the robot. Haocheng Sun et al. reported a seven-axis robot assistance system^13^. In there study the duration of operation, total blood loss, and drainage gradually decreased with the learning curve stage. However, it was a single-center study and had a relative small sample size. In our multicenter study, we firstly validated the accuracy and safety of a novel seven-axis robotic-assisted THA system. Current evidence suggests that the application of traditional six-axis robotic-assist systems leads to increased accuracy in positioning the prosthesis, especially the inclination and anteversion of the acetabular cup^22,23^. In the present study, the RAS group demonstrated 80.85% of the inclination and anteversion within the safe zone, which was significantly higher than that in the CON group, similar to the conclusion reported by Kong et al.^13,24^. Notably, when compared with the preoperative planning component position, the postoperative deviation was small. This indicated that the robot used in this study could achieve preoperative planning more accurately. As a seven-axis robotic arm system, this robotic arm can achieve better stiffness with the addition of a redundant degree of freedom to ensure surgical accuracy.

Soft-tissue tension following hip arthroplasty affects outcomes of patients^25^. Femoral offset, defined as the distance between the center of the femoral head and the femur’s anatomical axis, affects soft-tissue tension following THA. Current literature shows that the restoration of the optimal femoral offset improves the abductor lever arm and results in increased survivorship and reduced implant wear in THA^26,27^. However, Xiangjun Hu et al. showed that an increase of 2–3 mm in FO could improve the abductor and external rotator function following a THA^28^. Yang et al.^29^ believed that restored femoral offset achieved higher HHS score than in the decreased or increased femoral offset. In this study, we found that the femoral offset was restored more optimally in the RAS group compared to the CON group without significant differences. Firstly, The sample size of this article is relatively small. It may be the little recovery of femoral offset in the RAS group was not sufficient to cause significant functional scores compared with that in the CON group. Then, Significant correlations were found between the differences in acetabular offset, global femoral offset, femoral offset, and pain score in the study of Hirano et al^30^. In this study, only femoral offset was measured, which may not fully evaluate the “femoral offset”, which is one of the limitations of this study.

Postoperative LLD in cementless hip endoprosthesis remains a challenging issue. Patient dissatisfaction and litigation due to LLD are common, and a difference (usually lengthening) of > 7–10 mm is often evident and affects the functional outcome. LLD may warrant revision surgery due to clinical complaints of gait disorders and low back pain^31^. In addition to patient’s dissatisfaction after THA, LLD may increase stress on the superior part of the acetabular cup, elevate the risk of THA aseptic loosening, and accelerate osteoarthritis in adjacent joints^32^. Robotic-assisted THAs leading to accurate component positioning resulted in significantly less LLD compared with conventional THAs, which has also been proven previously^33,34^. But in the study, LLD was less in the RAS group compared to the CON group, with no significant difference between the two groups.

Since the three centers in this study were all famed joint surgery centers in China, and the surgeons involved in the study were highly experienced in THA, this could have led to the absence of any significant difference in the reconstruction of offset and LLD between the robotic-assisted and conventional THA. Perhaps the inclusion of surgeons of different levels in the study could more scientifically demonstrate the advantages of the new seven-axis robotic-assisted system over traditional surgery. And even for experienced surgeons, the implant position determined by personal experience remains significantly different from that determined during preoperative planning, indicating that the accuracy of the robot is noteworthy.

This study had some major limitations. Firstly, the sample size in the study was relatively small, and more cases were needed to validate this new robotic-assisted THA system. Secondly, no other robotic-assisted THA system was used as control group. It would be interesting to compare results using a 7-axis robot with using a 6-axis robot. But it is regrettable that it’s very difficult to execute relevant clinical studies, may not be realized until robot-assisted surgery really maturates. Thirdly, the follow-up time was relatively short, and long-term follow-up was needed to observe the improvement of clinical efficacy. At last, the three medical centers involved in this study were among the top levels in China, and it could be interesting to explore whether there was a significant difference in clinical outcomes between surgeons of different levels using the new seven-axis robotic-asistised THA system and those using traditional surgical methods.

In summary, the new robot-assisted THA system is a safe and effective system for THA.

## Data Availability

The datasets generated and/or analysed during the current study are not publicly available due to patient privacy but are available from the corresponding author on reasonable request.
